# ‘Seizure First Aid Training’ for people with epilepsy who attend emergency departments, and their family and friends: study protocol for intervention development and a pilot randomised controlled trial

**DOI:** 10.1136/bmjopen-2015-009040

**Published:** 2015-07-24

**Authors:** AJ Noble, AG Marson, C Tudur-Smith, M Morgan, DA Hughes, S Goodacre, L Ridsdale

**Affiliations:** 1 Department of Psychological Sciences, University of Liverpool, Liverpool, UK; 2 Department of Molecular and Clinical Pharmacology, University of Liverpool, Liverpool, UK; 3 Department of Biostatistics, University of Liverpool, Liverpool, UK; 4 Institute of Pharmaceutical Science, King's College London, Liverpool, UK; 5 Centre for Health Economics & Medicines Evaluation, Bangor University, Bangor, UK; 6 School of Health and Related Research, University of Sheffield, Sheffield, UK; 7 Department of Basic and Clinical Neuroscience, King's College London, London, UK

**Keywords:** ACCIDENT & EMERGENCY MEDICINE, EDUCATION & TRAINING (see Medical Education & Training), QUALITATIVE RESEARCH, REHABILITATION MEDICINE, THERAPEUTICS

## Abstract

**Introduction:**

People with chronic epilepsy (PWE) often make costly but clinically unnecessary emergency department (ED) visits. Offering them and their carers a self-management intervention that improves confidence and ability to manage seizures may lead to fewer visits. As no such intervention currently exists, we describe a project to develop and pilot one.

**Methods and analysis:**

To develop the intervention, an existing group-based seizure management course that has been offered by the Epilepsy Society within the voluntary sector to a broader audience will be adapted. Feedback from PWE, carers and representatives from the main groups caring for PWE will help refine the course so that it addresses the needs of ED attendees. Its behaviour change potential will also be optimised. A pilot randomised controlled trial will then be completed. 80 PWE aged ≥16 who have visited the ED in the prior 12 months on ≥2 occasions, along with one of their family members or friends, will be recruited from three NHS EDs. Dyads will be randomised to receive the intervention or treatment as usual alone. The proposed primary outcome is ED use in the 12 months following randomisation. For the pilot, this will be measured using routine hospital data. Secondary outcomes will be measured by patients and carers completing questionnaires 3, 6 and 12 months postrandomisation. Rates of recruitment, retention and unblinding will be calculated, along with the ED event rate in the control group and an estimate of the intervention's effect on the outcome measures.

**Ethics and dissemination:**

Ethical approval: NRES Committee North West—Liverpool East (Reference number 15/NW/0225). The project's findings will provide robust evidence on the acceptability of seizure management training and on the optimal design of a future definitive trial. The findings will be published in peer-reviewed journals and presented at conferences.

**Trial registration number:**

ISRCTN13 871 327.

**Strengths and limitations of this study**

- This will be the first study to develop and pilot seizure first aid training for people with epilepsy who frequently visit hospital emergency departments (EDs), and their carers.
- The intervention will be developed so that it closely aligns with service users' needs and preferences in order to maximise its acceptability and benefit.
- Its method of delivery and low cost could mean it holds the potential to be generalisable across the health service and sustainable.
- The follow-up period in the pilot randomised controlled trial will be 1 year and the data on the primary outcome measure, namely subsequent ED use, will be collected using objective hospital data.
- We expect that the pilot will provide robust estimates to inform the optimal design of a future definitive trial.

## Introduction

### Emergency hospital use for epilepsy

With a prevalence of up to 1%,^1^ epilepsy is one of the most common brain disorders in the UK.^2^ As well as having significant implications for the lives of patients,^3^ epilepsy also has important societal impacts.^4^ ^5^ Studies show that one of these is the cost of providing emergency care.^6–8^

In the UK, one fifth of people with epilepsy (PWE) visit hospital emergency departments (ED) each year for seizures,^6–8^ with rates being highest in socially deprived areas.^9^ ^10^ In England in 2012/2013, the cost of providing emergency care for epilepsy was >£56 million.^11^ One reason it is so high is because half of the PWE visiting EDs are admitted to hospital;^8^ ^12–14^ indeed, 85% of admissions for epilepsy occur on this unplanned basis.^15^ Readmissions further drive costs up;^16^ ≥60% of PWE reattend ED within 12 months.^17^

Seeking emergency care for epilepsy can be important, even life-saving. However, most ED visits by PWE are clinically unnecessary. The UK-wide National Audits of Seizure Management in Hospitals (NASH)^18^ ^19^ found that most visits were by people with known rather than new epilepsy and most had experienced uncomplicated seizures. Guidelines are clear that, with the correct training, such seizures can be safely managed by patients and their families within the community.^20–22^ Factors beyond clinical need have been identified as often being important in determining whether an emergency admission occurs, which most likely explains why so many visits to the ED by people in whom the diagnosis is clear and who have made a full recovery still end in hospital admission.^8^ ^23^

Reducing unnecessary emergency visits to hospital by PWE has been identified as one way that resource-limited health services can generate savings.^24^ Reducing emergency visits is also important for service users. ED visits can be inconvenient, distressing and do not typically lead to extra support.^12^ There may even be iatrogenic harm, such as that associated with unnecessary intravenous cannulations.^25^

### Reasons for emergency hospital use

It has been challenging to know how to reduce emergency visits for epilepsy,^26^ not least because the reason/s for them were unclear. The association between seizure frequency and ED use had, for example, been found to only be modest in size and seizure type had not proved a robust predictor.^6^ ^27–29^

However, a recent mixed-methods study has brought clarity to the issue.^11^ It suggests that what is often key in determining whether someone visits the ED for seizures is not necessarily clinical need, but confidence in seizure management.^30^ ^31^ Eighty-five adults with epilepsy were prospectively recruited from UK EDs and interviewed. Patients fell into two groups. In the first, there were patients who reported high levels of confidence. Their views closely aligned with seizure first aid guidelines. They had typically visited the ED only once in the previous 12 months.

In contrast, patients in the second group did not feel confident managing seizures and had typically made ≥2 ED visits in the prior year. They feared seizures, including the possibility of death. This led them to call for an ambulance when they believed they were about to have, or had had, a seizure. Despite having diagnosed epilepsy for ∼10 years, they said they had not received sufficient information about epilepsy.

Quantitative results from the project reinforced what patients said. Regression analyses identified that it was a patient's score on a measure of perceived ‘mastery’ over their epilepsy which significantly predicted how many ED visits they made over the subsequent 12-months rather than seizure frequency.^29^ There was also evidence of poor first aid knowledge. One third of the sample incorrectly stated that it was always necessary to call a doctor or ambulance if a person with epilepsy has a seizure, even if it occurs without complications.^17^ Only 11% of the wider epilepsy population believe this (S Jarvie. *Self perception and psychosocial functioning in people with intractable epilepsy* [PhD thesis, Unpublished data]. University of Glasgow, 1993).

The above findings are in keeping with prior evidence. Coping with life in the context of epilepsy requires PWE to learn and adopt specific self-management behaviours to prevent seizures and manage consequences. It is known, however, that PWE typically receive little support from health services in learning to self-manage.^32–35^ One consequence is that knowledge about epilepsy among patients can be poor, especially in those with low education.^36^ ^37^

Another important finding from the interviews conducted for the project was that when seizures occurred, responsibility for patient care and the decision to seek emergency care could be delegated to family or friends. When these persons were confident in seizure management and been correctly informed, the patient would visit the ED only under certain circumstances. However, when they were not, they would often seek emergency medical care, regardless of clinical need: One said, “[I was] just worried because I don't know anything about epilepsy… I mean I only know the bad things, I know it can be quite serious… I know you can die… I was so worried I decided just to ring an ambulance…better safe than sorry.”^30^ This accords with evidence that most seizures leading to the ED appear to occur within patients’ homes and are often witnessed.^13^ ^38^

### Need for seizure first aid training

On the basis of the evidence presented, PWE who frequently visit the ED might benefit from a self-management intervention that improves their own and their informal carers’ confidence and ability in managing seizures and empowers them to be able to tell others from their wider support network about first aid.

No epilepsy self-management intervention is available which focuses on seizure management, or on those attending the ED, and none systematically involves carers.^39–41^ That such an intervention might improve seizure management skills is, however, supported by the broader literature. Studies on general first aid show that even brief interventions can improve skills in a variety of groups.^42–46^ Evidence from asthma studies is also important.^47–49^ Boyd *et al*^47^ reviewed 17 randomised controlled trials (RCT) of educational interventions for children (and parents) at risk of asthma-related ED attendances. The interventions led to a 37% reduction in the relative risk of reattendance at the ED and a 21% reduction in subsequent hospital admissions.

### Aims

We describe here a project to develop and then pilot a seizure first aid training intervention for PWE who frequently visit the ED and their carers (protocol V.1.1, 31/3/15). This project will be completed in the Merseyside area of North-West England.

Rather than creating an entirely new intervention, it will be developed by adapting a promising seizure management course that already exists to address the needs of PWE visiting the ED. The course titled ‘Epilepsy awareness and seizure management’ has been offered on a small scale within the third sector to people from a variety of backgrounds, including patients, teachers and care home staff by the UK charity, Epilepsy Society. The society has offered the course since 1998 and given us permission to adapt it. It has not been formally evaluated, but aims to increase participants’ confidence in seizure management.

Changes to the existing course will be required since it was developed for delivery to a narrower, fee-paying group. It was not created for delivery within the health service, nor for PWE who visit EDs who can be particularly challenged by epilepsy and may have lower education.^9^ ^17^ ^27^ ^36^ ^37^ Once adapted, a pilot RCT shall be completed. A pilot is required to address uncertainties about the optimal design of a full RCT.^50^ These include intervention acceptability, likely effect, as well as participant uptake and retention.

Accordingly, the project's objectives are to:

1. Optimise the content, delivery and behaviour change potential of the existing course for PWE attending the ED, and their informal carers. The resulting adapted package will be named Seizure First Aid Training.
2. Conduct a pilot RCT of Seizure First Aid Training versus Treatment As Usual (TAU) alone to estimate likely recruitment, consent and follow-up rates in a future definitive trial.
3. Test acceptability of randomisation to participants.
4. Calculate estimates of the annual rate of ED visits in the control group and the likely dispersion parameter to inform the sample size calculation of a future RCT.
5. Conduct an analysis of the cost of implementing the Seizure First Aid Training programme.

## Methods and analysis

### Part A: Intervention development (months 1–8)

#### Design

**Figure 1:**
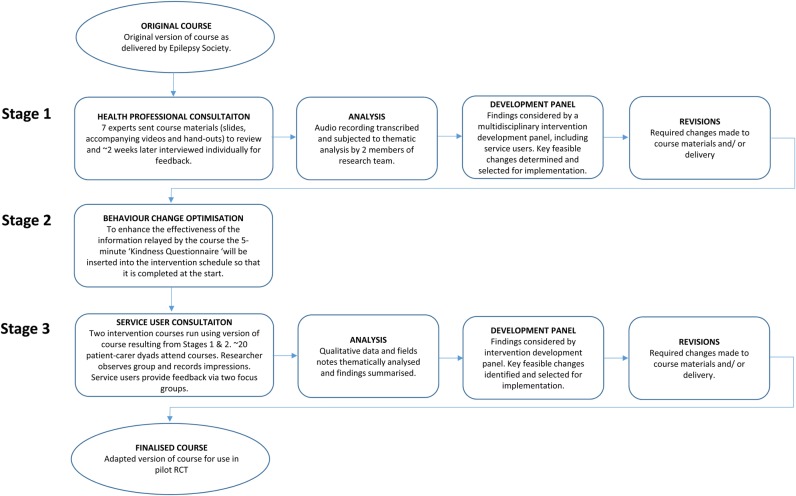
Intervention development process.

To adapt the existing course, three stages will be completed (figure 1).

##### Stage 1: Consultation with main professional groups

Leading representatives from the professional groups supporting PWE will review the course materials and be interviewed. Consensus exists on seizure first aid^22^ and so, where expertise permits, representatives will be asked to identify changes needed to ensure accuracy, as well as suggestions for improvement.

##### Stage 2: Optimisation of behaviour change potential

A significant component of the intervention consists of providing information about epilepsy, its manifestations and first aid. This will highlight to some participants that their past behaviour conflicts with medical guidance. This could be construed as a threat to self-integrity.^51–53^ Self-Affirmation theory states that people are motivated to maintain self-integrity.^53^ Accordingly, threats to the self can be defensively processed (eg, motivated scepticism, unrealistic optimism). To mitigate against this, Reed and Aspinwall's self-affirmation^54^ ‘Kindness Questionnaire’ will be inserted to the beginning of the intervention. Completing such an exercise is known to reduce resistance to threatening or dissonant information and increase behaviour change.^55^ ^56^

##### Stage 3: Consultation with service user representatives

Health professionals and PWE may have different views of support needed.^57^ For this reason, two courses using the initial adaptation of the course will be run with patient-carer dyads and focus groups will explore users’ views of the intervention, its content, the facilitator, scheduling and acceptability.

### Participants

For Stage 1, representatives from neurology, emergency medicine, the ambulance service, nursing, general practice, user groups and health care commissioning based within the UK have been identified, approached and will be asked to provide informed consent. For Stage 3, user groups within the Merseyside area will help identify and recruit dyads. PWE will be eligible to participate if they are aged ≥16 and have visited ED in the past 2 years. The full inclusion/exclusion criteria are in table 1.

**Table 1:** Participant inclusion and exclusion criteria

Study part	Inclusion criteria	Exclusion criteria
*Part A—Intervention development*		
Patients	Established diagnosis of epilepsy (≥1 year) All epilepsy syndromes and all types of focal and generalised seizures Currently being prescribed antiepileptic medication Age ≥16 years (no upper age limit) Have visited ED in the past 2 years for epilepsy (as reported by the patient) Live in the North West area of England Able to provide informed consent and participate in intervention in English	Acute symptomatic seizures related to acute neurological illness or substance misuse Severe current psychiatric disorder (eg, acute psychosis) or life-threatening medical illness
Carers	A significant other to the patient (eg, family member, friend) whom the patient identifies as providing informal support Age ≥16 years (no upper age limit) Live in the North West area of England Able to provide informed consent and participate in intervention in English	Severe current psychiatric disorder or life-threatening medical illness
*Part B—Pilot RCT*		
Patients	Established diagnosis of epilepsy (≥1 year) All epilepsy syndromes and all types of focal and generalised seizures Currently being prescribed antiepileptic medication Age ≥16 years (no upper age limit) Visited an ED for epilepsy on ≥2 occasions within the previous 12 months (as reported by patient) Live within 25 miles of any of the ED recruitment sites Able to provide informed consent, participate in intervention and independently complete questionnaires in English	Actual or suspected psychogenic non-epileptic seizures alone or in combination with epilepsy Acute symptomatic seizures related to acute neurological illness or substance misuse Severe current psychiatric disorders (eg, acute psychosis) or life-threatening medical illness Enrolled in other epilepsy related non-pharmacological treatment studies
Carers	A significant other to the patient (eg, family member, friend) whom the patient identifies as providing informal support Age ≥16 years (no upper age limit) Lives in the North West area of England	Severe current psychiatric disorders or life-threatening medical illness Enrolled in other epilepsy related non-pharmacological treatment studies

While efforts will be made to maximise the recruitment of patient-carer dyads, patient participants will be permitted to take part without a carer. Carers will not, however, be able to take part in this part of the project without a patient partner having at least consented to take part in the study. Up to 90% of PWE can identify an informal carer.^58^

ED, emergency department PWE, people with chronic epilepsy; RCT, randomised controlled trial.

### Intervention in its current form

The existing course lasts 3 h. It is delivered to groups of 10–20 people by an educational facilitator. It covers eight topics (table 2) and emphasises how most seizures are self-limiting. It seeks to provide participants with a practical understanding of when seizures do, and do not, require emergency treatment.

**Table 2:** Topics covered by existing version of a seizure first aid training course

Topic	Details
1.	What is epilepsy? Myths and truths about epilepsy are discussed, and a simple explanation is provided of what happens in the brain to produce seizures
2.	Different causes of epilepsy and seizure triggers
3.	Diagnosis: important diagnostic tools are discussed
4.	Detailed discussion of seizure types, their effects, and how to manage each of them, including when to call an ambulance and demonstration of the recovery position. This includes video clips showing different types of seizures, with PWE and health professionals discussing them
5.	Status epilepticus
6.	Treatments: medication and side effects
7.	Risk management and support needs
8.	Sources of further information: addresses of organisations offering assistance and information

Course materials include standardised slides, videos and an information pack. The pack provides participants with a permanent record, as well as space for notes to promote active processing. Participants are encouraged to share experiences and ask questions. The course consists of a number of components and so is a complex intervention.^50^

Facilitators typically have a nursing or social care background, experience of working with PWE and follow a local Epilepsy Society training programme in order to deliver the course.

As well as holding the potential to increase seizure management confidence and lead to fewer unnecessary ED visits, further justification for adapting this particular course is that its delivery method could be generalisable. Specialist epilepsy nurses and physiologists have been asked to deliver broader self-management interventions.^39^ ^59^ Such staff are, however, not widely available.^8^ ^60^ ^61^ The epilepsy voluntary sector is particularly well developed,^26^ ^62^ ^63^ and so commissioning third sector organisations to deliver seizure-management training could help avoid shortfalls. The Epilepsy Society, for example, currently has a bank of 12 educational facilitators located around England able to deliver its courses, and it already has a role in providing information materials for hospital clinics. The model could also be financially sustainable, with the Epilepsy Society charging only £40 to attend its course.

Another reason for adapting this particular course is that its content and format broadly align with service users’ preferences, thus increasing its likely acceptability. Studies show that PWE tend to want short, face-to-face self-management courses, and for them to be delivered by persons with knowledge or experience of epilepsy.^64–68^

### Part B: Pilot RCT (months 9–38)

#### Design

**Figure 2:**
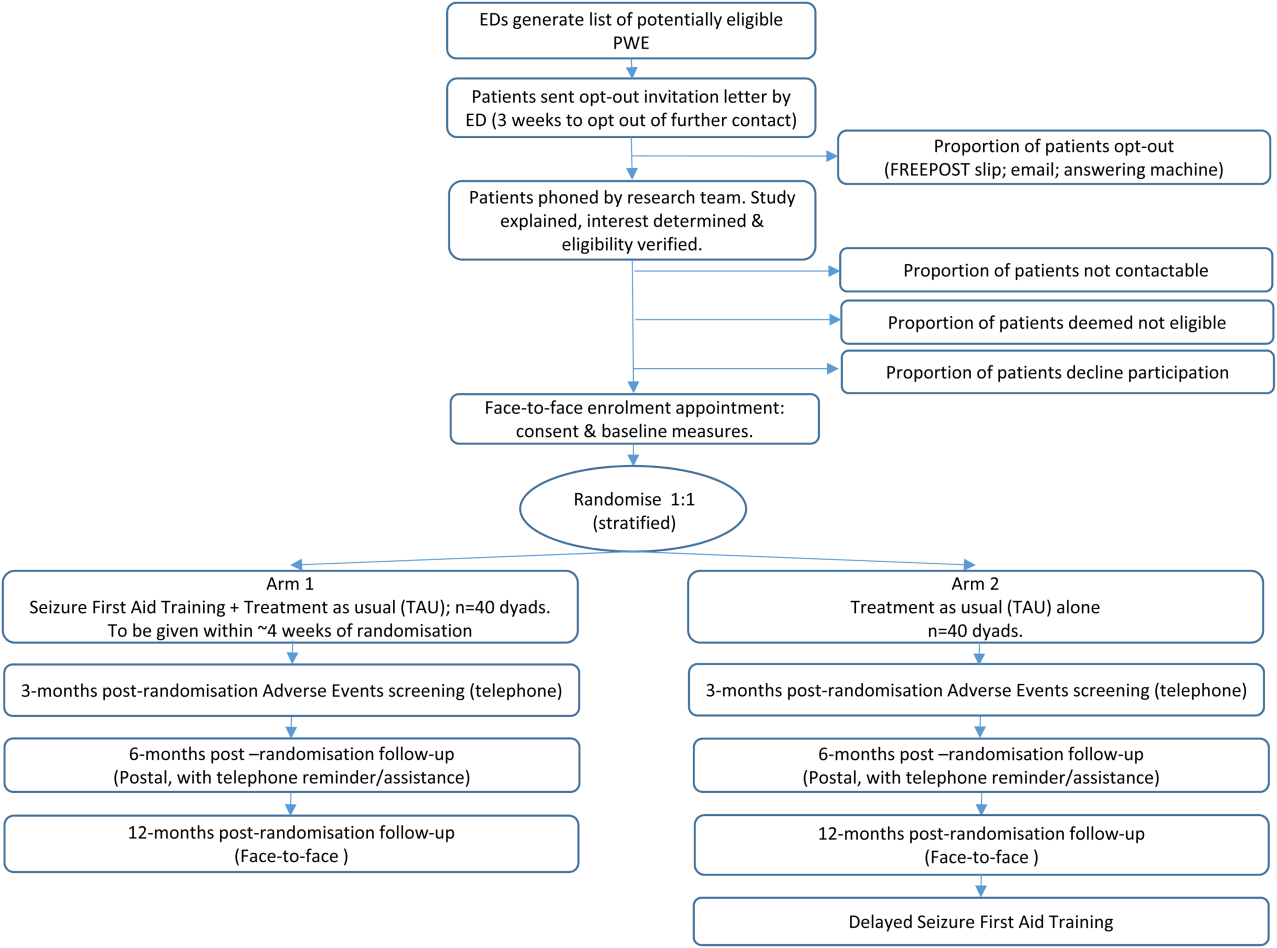
Design of the pilot trial phase of the project. ED, emergency department PWE, people with chronic epilepsy.

Using the adapted seizure management course, a multicentre, external pilot RCT will be completed with PWE aged ≥16 years who have visited the ED in the prior 12 months for epilepsy on ≥2 occasions, along with one of their family members or friends who have an informal caring role. Patients and carers will be followed up for 12 months. The participants and data flow in the study are shown in figure 2.

### Participants

The EDs of three Merseyside NHS hospitals have been recruited to identify potential participants. They serve a population of ∼830 000 people, within which the prevalence of adult epilepsy is 0.98%.^69^ This population features high levels of social deprivation^70^ ^71^ and rates of emergency admissions for epilepsy that are among the highest.^9^

To identify eligible patients, the EDs will complete searches of their electronic attendance records for persons with a presentation/discharge code in the prior 12 months that is indicative of epilepsy. The eligibility criteria are provided by table 2.

Invitation letters from the local consultant will be sent to all ostensibly eligible patients. Those not opting out of further contact within 3 weeks will be phoned by the research team which will confirm interest, verify eligibility and, if applicable, arrange an initial meeting. At that meeting, consent will be obtained and baseline data collected from the patient and their nominated family member or friend.

### Randomisation and concealment

Computer-generated randomisation of patient-carer dyads will be completed remotely by the Clinical Trials Research Centre (CTU) at the University of Liverpool following consent and completion of the baseline measures. The unit of randomisation will be the individual participant and randomisation will be 1:1 between the intervention and the control groups, stratified by factors which at this stage will not be disclosed to prevent treatment prediction.

The results of the allocation will be concealed from the trial statistician and researchers responsible for consent and data collection. A study administrator will liaise with patients (and carers) to arrange attendance at the intervention. Participants will be asked not to inform the research team of their treatment allocation and we shall test the success of blinding.

### Interventions

#### Intervention arm: seizure first aid training (+TAU)

The intervention's exact content will be determined in Part A of the project. It is anticipated, however, that it will continue to last ∼3 h, with no additional booster sessions, and be delivered to groups of ∼10 patient-carer dyads by local Epilepsy Society educational facilitators. Courses will be delivered within educational rooms at the local hospitals from where recruitment occurred. Both patients and carers will be expected to participate actively in the course. Participants will each be provided with an information pack. These will include copies of the course material, certificates of attendance, epilepsy identification cards, wallet sized first aid instruction cards and contact details for further information.

#### Control arm: TAU only

The active intervention will be compared to TAU alone by the PWE's normal care team. No restrictions will be placed on the services TAU participants can access.

In the UK, there is no accepted care for those with established epilepsy who have visited an ED.^26^ All PWE are, however, expected to have a medical review of their epilepsy at least yearly by a generalist or specialist. When seizures are not controlled or treatment fails, it is expected that a patient will be referred to secondary or tertiary services.^26^ The UK's NASH showed that EDs initiate referral to neurology for only a third of PWE attending the ED.^18^ ^19^

Delayed access to the Seizure First Aid Training for control participants is being used as a recruitment/retention incentive. These courses will be run once all retained patient and carer participants from both arms have completed their final follow-up assessments. The TAU group will only contribute outcomes to the trial data set under the TAU condition.

### Outcomes and outcome measures

#### Primary

The proposed primary outcome measure for a future definitive trial is the number of epilepsy-related ED visits made over the 12 months following randomisation by patient participants. This will be measured in the pilot using routinely collected hospital data. The NHS’s Hospital Episode Statistics system provides a record of an individual's use of all EDs in England and data will be extracted from it to provide information on individual participants’ use of the ED at baseline and over follow-up. While this system is increasing in sophistication, it does not currently have a code to indicate visits related specifically to epilepsy. However, in order to increase specificity and provide a more reasonable estimate of such visits, we shall utilise a broader code that does exist within the system so as to identify only visits related to a central nervous system condition (excluding stroke).

#### Secondary

Over the course of the trial, patient and carer participants will be required to each complete three sets of questionnaires (table 3) either in a face-to-face interview with a research worker (at baseline and at 12-month follow-up) or through the post (at 6-month follow-up). To encourage continued participation, we will offer each participant a £10 voucher on completion of each assessment.^86^

**Table 3:** Self-reported secondary outcome measures by assessment and participant type

Outcome	Participants	Measure	Items (n)	Baseline	6-month	12-month
Knowledge and fear of seizures	Patients; carers	*Epilepsy Knowledge and Management Questionnaire—Fears subscale*^72^	5	✓	–	✓
Knowledge of what to do when faced with a seizure	Patients; carers	*Items from Thinking About Epilepsy Questionnaire*^73^	3	✓	–	✓
Confidence managing seizures/epilepsy	Patients; carers	*Epilepsy Mastery Scale*^74^ (P); *Parents Response to Child Illness Scale—Condition Management subscale*^75^(C)	6	✓	✓	✓
Quality of life	Patients	*Quality of Life in Epilepsy Scale-31*^76^	31	✓	✓	✓
Distress	Patients; carers	*Hospital Anxiety and Depression Scale*^77^	14	✓	–	✓
Seizure control	Patients	At baseline, *Thapar's Seizure Frequency Scale* for the prior 12 months.^78^At follow-up, patients will be asked for number of seizures (of any type) since the last assessment and dates of the first and most recent*	1	✓	✓	✓
Felt Stigma	Patients; carers	*Stigma of Epilepsy Scale*^79^ ^80^	3	✓	–	✓
Burden	Carers	*Zarit Caregiver Burden Inventory*^81^	22	✓	✓	✓
Activation	Patients; carers	*Patient Activation Measure*^82^	13	✓	–	✓
Health economics	Patients	*Client Service Receipt Inventory*^83^ and *EQ-5D* 84	13	✓	–	✓
Feedback on trial participation	Patients; carers	Adapted from Magpie Trial^85^	3	–	–	✓

*To assist patients to be able to provide this information, they will be offered a seizure diary at their baseline appointment.

Secondary measures will be based on participant self-report and be used to help estimate whether the intervention leads to changes including improved quality of life, confidence managing seizures, knowledge of seizure first aid, as well as reductions in fear of seizures. Patient participants will also be asked to self-report on their service use, including of ED and ambulances. A full list of the measures to be used is provided in table 3.

Participants will be requested to complete the measures prior to randomisation and then 6 and 12 months postrandomisation. Baseline and 12 month follow-up measures will be collected in face-to-face sessions by a research worker, blind to treatment allocation. Estimated completion time is 1 h. An abbreviated assessment will occur at 6 months. For it, participants will be posted a set of questionnaires for completion.

### Statistical analysis

Since this is a pilot RCT, a formal power calculation is not appropriate; the study will not be powered to detect a clinically meaningful difference in the primary outcome between the treatment groups. Rather, we aim to generate the following: estimates of eligibility, consent, recruitment and retention rates and speed of recruitment; and estimates of completion rates of study assessment tools and rates of unblinding. To accurately inform a sample size calculation for a future definitive trial, estimates of the ED event rate and dispersion parameter will also be provided, along with summary statistics measuring the effect of the intervention on the primary and secondary outcome measures and the precision of such estimates.

Forty patients in each treatment arm will provide the above estimates with adequate precision. In particular, with a sample size of 80, we will be able to estimate an overall dropout rate of 25% (approximate rate experienced by similar studies^11^ ^39^ ^40^) to within a 95% CI of ±10% and a participation rate of 20% from an assumed 400 patients to within a 95% CI of ±4%. Assuming that the ED data at 12 months is not available for 25% of patients, outcome data from 60 patients would still allow robust estimation of the ED rate and dispersion parameter.^87^ ^88^

### Minimising bias

PWE will attend the course outside of their routine clinic appointments and we do not expect transfer of intervention-related knowledge (and therefore contamination of the TAU group) between those in the intervention and control arms at a single site. Bias will be further minimised by restricting access to and availability of the intervention materials. When participants are asked to self-report on their service use, we shall also ask those in the control group whether they accessed any elements of the intervention.

## Discussion

### Ethics and oversight

Monitoring by an independent Study Steering Committee (SSC) will help to ensure that the rights, safety and well-being of the participants are the most important considerations. Compliance with Good Clinical Practice and scientific integrity will be managed by the study management team through regular and ad hoc meetings. Guidelines from the project's funder indicates that a Data Monitoring and Ethics Committee is not required (http://www.nets.nihr.ac.uk/__data/assets/…/ssc-and-dmec-checklist-june13.doc).

Patients’ experience of unexpected serious adverse events (SAE) within the trial phase will be monitored by asking them to complete a standardised SAE checklist. Patients will be asked to complete it by phone at 3 and 6 months postrandomisation, and in person at the 12-month follow-up appointment. A neurologist will assess unexpected SAEs, and the approving ethics committee and sponsor will be informed within 15 days of any SAEs that are judged to be related to participation. Given the population, the following will not be deemed as unexpected SAEs: epileptic seizures with or without injury; visits to the ED where the stay lasts <24 h; side effects of antiepileptic medication; or diagnosis of a comorbid psychiatric condition.

For the trial phase, the CTU will provide regular reports on data quality to ensure the integrity of randomisation, to monitor the level of missing data and the timeliness of data entry and to check for illogical or inconsistent data. Data collection procedures will be monitored and source data verification against the paper data collection forms undertaken at regular intervals.

Recruitment for studies involving serial assessments can be low. Owing to the social impact of epilepsy and its comorbidity, recruitment of PWE can also be challenging.^89^ For the project, only patients and carers providing signed, informed consent will be able to participate. However, to maximise uptake, we shall utilise an ‘opt out’ method of inviting patients, rather than the traditional ‘opt in’ approach. Specifically, a letter, signed by the ED consultant, will be sent to all ostensibly eligible individuals explaining the study and inviting them to participate. Persons will be informed that unless they opt out (by email, telephone or using a FREEPOST response slip) within 3 weeks, it will be taken that they are interested in being telephoned by the research team with further information.

The approach is ethically justifiable.^90^ It significantly increases participation rates,^91^ reduces the likelihood of a biased sample of participants being recruited,^92^ and is more cost-effective compared to an ‘opt in’ approach.^93^ We are currently using a version of the opt-out approach for another trial with PWE and the approach has proved largely acceptable.^59^

### Dissemination

Despite the demonstrated need, there has been inadequate attention given to implementing and evaluating interventions to increase seizure management confidence in PWE who visit ED and their informal carers. We have described a study that will develop such an intervention for use within the UK health service. The trial phase aims to provide design information for a future definitive trial and quantitative estimates of the impact of the intervention on patients in terms of its clinical and cost-effectiveness. We will use the findings to inform the development of a future definitive RCT, with the ultimate goal of reducing clinically unnecessary ED use and improving the well-being of PWE who visit ED and their carers.
